# A Difference in the Count

**Published:** 1881-02

**Authors:** 


A Difference in the Count.—Dr. James A. Steuart, Health Commissioner of this city, has sent a letter to Dr. T. J. Turner, of the National Board of Health, declining to prepare the mortuary reports of Baltimore by the last census. Some time since the National Board of Health notified Dr. Steuart that the death rate and health reports generally must hereafter be made up according to the estimated population of the city by the last census. This the health department refused to do, claiming that the census is grossly incorrect and Baltimore's population misrepresented. Dr. Steuart has therefore notified the National Board that he will not permit the figures of the Baltimore Board of Health to be changed in the reports.

This action of Dr. Steuart will meet the approval of every descendant of the patriots who aided in achieving the independence of the States, which established a general government of limited powers to do what was inconvenient or impracticable for them to do within themselves, and who has not sold his birthright for a mess of pottage.

